# Psychometric properties of the newly developed Physician Teaching Self-Efficacy Questionnaire (PTSQ)

**DOI:** 10.1186/s12909-016-0764-4

**Published:** 2016-09-22

**Authors:** Christoph Dybowski, Levente Kriston, Sigrid Harendza

**Affiliations:** 1 Department of Internal Medicine, University Medical Center Hamburg-Eppendorf, Hamburg, Germany; 2 Department of Medical Psychology, University Medical Center Hamburg-Eppendorf, Hamburg, Germany

**Keywords:** Clinical teaching, Motivation, Self-efficacy, Teaching experience, Teaching involvement, Teaching quality, Undergraduate medial education, Validation

## Abstract

**Background:**

High teaching quality and students’ corresponding learning progress are the most important indicators of teachers’ work performance. Theory and numerous empirical studies indicate that self-efficacy, a person’s belief in her or his ability to accomplish a task, is an important predictor of work performance. Accordingly, it can be assumed that teaching self-efficacy also influences teaching performance and students’ learning progress with regard to physicians who teach in undergraduate medical education. Therefore, the aim of this study was to develop and validate an instrument measuring clinical teaching self-efficacy in physicians.

**Methods:**

We developed 16 items reflecting physicians’ beliefs to provide high quality clinical teaching when facing regularly occurring critical teaching situations. These constitute the Physician Teaching Self-Efficacy Questionnaire (PTSQ). For its validation, we used data from a sample of 247 physicians from internal medicine and surgery at six German medical faculties. Regarding factorial validity, we performed exploratory structural equation modelling (ESEM) as well as confirmatory factor analysis (CFA). Regarding criterion validity, correlations with the scales of the Physician Teaching Motivation Questionnaire (PTMQ), teaching experience and perceived teaching involvement were calculated. Additionally, we conducted the same analyses with a short 6-item version.

**Results:**

ESEM delivered evidence for a three-factor structure with a superordinate general factor, which was confirmed by local and global fit indicators in CFA (RMSEA = .055, TLI = .939, SRMR = .048, CFI = .948). We identified the following three subfactors: teaching self-efficacy with respect to self-regulation, dyadic regulation involving students, and triadic regulation involving students and patients. Internal consistencies indicated acceptable to excellent reliability for all scales (Cronbach’s alpha = .77–.90). Theory-consistent correlations with the PTMQ scales, teaching experience, and teaching involvement confirmed criterion validity. Besides excellent global fit, the short version of the PTSQ also fulfilled all other validity criteria.

**Conclusions:**

The PTSQ is a valid instrument to assess physicians’ clinical teaching self-efficacy. It could be used in faculty development programmes and for educational research. The short version could be used in situations that are time-critical for physicians in order to ensure high response rates.

**Electronic supplementary material:**

The online version of this article (doi:10.1186/s12909-016-0764-4) contains supplementary material, which is available to authorized users.

## Background

Within the context of medical education, self-efficacy has been investigated almost exclusively as a student variable and has been found to predict the academic performance of medical students [1–3]. This finding is consistent with studies from non-medical educational contexts [4–7] and supports the theoretical assumptions regarding self-efficacy. Albert Bandura (1977) introduced the concept of self-efficacy as an essential part of the social cognitive theory (SCT) and defined it as “the belief in one’s capabilities to organize and execute the courses of action required to manage prospective situations” [8, 9]. According to SCT, self-efficacy has an indirect effect on motivation through several cognitive processes and cognition-guided behaviors: “Self-efficacy beliefs contribute to motivation in several ways: They determine the goals people set for themselves; how much effort they expend; how long they persevere in the face of difficulties; and their resilience to failures” [9]. These cognitions and behaviors furthermore make an impact on performance: “Strong perseverance contributes to performance accomplishments” [9]. In meta-analyses, substantial evidence for the positive relationship between self-efficacy beliefs and work performance was gathered [10–12].

If students’ learning progress depended on physicians’ teaching performance, physicians’ teaching self-efficacy might play an important role in predicting teaching quality. However, to our knowledge, no studies on the impact of physicians’ teaching self-efficacy on teaching quality have been conducted to date and no instruments have been published which measure physicians’ teaching self-efficacy in undergraduate medical education. In this context, only student self-efficacy scales have been developed, for example for palliative care [13], interaction with parents [14], exhibiting patient-centered behaviors [15], and competencies based on the CanMEDS framework [16]. In non-medical contexts, self-efficacy scales have been developed for a wide range of subjects and contexts including schoolteachers’ self-efficacy [17, 18] and work self-efficacy in general [19, 20]. A positive impact of teaching self-efficacy on students’ academic achievement has been demonstrated [21–23]. Furthermore, higher teaching self-efficacy was found to be associated with more persistent and less critical behavior [24] and striving for better didactic methods [25]. Instruments measuring general work self-efficacy cannot be applied to medical faculty staff, as teaching usually only constitutes one work task for physicians besides patient care and research. The available instruments for measuring schoolteachers’ self-efficacy are context dependent and cannot be applied to teaching physicians at university hospitals.

Another theory that implies an effect of self-evaluation on motivation and on performance is the self-determination theory (SDT) [26]. It proposes a multidimensional view of motivation and distinguishes between several types of motivation depending on the level of involved self-regulation. Furthermore, it postulates that more autonomous types of motivation give rise to higher effort in actions at which the motivation is targeted, and that the self-evaluation of competence is among the influential factors of motivation. While both constructs, self-efficacy according to SCT and perceived competence according to SDT, aim at the belief in one’s ability to master a certain task, perceived competence in SDT stresses the personal meaningfulness and importance of a task on which the satisfaction of a person’s needs depends. However, we hypothesize that both constructs share a sufficiently big overlap so that self-efficacy has a very similar effect on motivation as perceived competence has according to SDT.

The aim of our study was to develop and validate a comprehensive teaching self-efficacy questionnaire tailored to physicians involved in undergraduate medical education. We defined teaching self-efficacy as physicians’ beliefs in their capability to carry out the actions necessary to provide high quality teaching to medical students, affecting both teaching motivation and teaching involvement. Based on SDT, we assumed that higher teaching self-efficacy is associated with more autonomous types of teaching motivation and higher teaching involvement.

## Methods

In classical conceptualizations, validity has been defined as three separate types, content, construct and criterion validity [27]. For this study, we followed modern conceptualizations which define validity as a unitary concept with construct validity as a core and recommend to derive validity evidence from several sources, including assessments of content validity, the response process, the internal structure of the instrument, its relationships to other variables, and its consequences [28]. In order to fulfil these requirements, we constructed the items precisely to ensure content validity. The response process was analysed with participants of the target group. The internal structure was assessed with respect to dimensionality and scale reliabilities. The relationship to other variables was assessed in terms of concurrent criterion validity. The consequences of testing are discussed in the context of the fields of usage for our instrument.

### Development of the physician teaching self-efficacy questionnaire (PTSQ)

For item development, we adhered to Bandura’s guidelines for the construction of scales measuring self-efficacy [29]. Bandura (2006) stressed the importance of tailoring items to specific situations and specific tasks, as only specific instruments have predictive validity for outcomes related to these situations or tasks [29]. Furthermore, he stated that difficulties are necessary to be adequately presented in the items, as in situations with a very low level of difficulty “everyone is highly efficacious” and the scale would not differentiate between high and low self-efficacy.

Based on previous findings [30–35], we developed 16 items including typical critical situations regularly faced by physicians involved in clinical teaching, e.g. time pressure, language problems with patients, difficulties with patient selection, short-term allocation of teachers to lessons or demotivated students. Fourteen of these items are introduced by “Even if…” or a similar phrase, followed by an obstacle to teaching and a statement that the respondent is nevertheless able to provide different aspects of high quality teaching. Criteria for high teaching quality were taken from a review [36]. Examples are “Even if I am under time strain, I am able to concentrate and provide a well-structured lesson” and “Even if students seem tired or demotivated, I manage to make them enthusiastic about the lesson”.

There are two deviations from this structure. Firstly, two items only provide a statement about a competence without a teaching obstacle (“I am a very good role model for the students regarding my interaction with patients” and “I am very good at adapting my teaching to different degrees of prior knowledge in a student group”). Secondly, five items included a general statement on overall teaching quality, as we wanted to retain the explicit subjectivity of the construct, for example “Even if I am in a bad mood or stressed, I teach well”.

As an introduction to the questionnaire, we used the following: “One can feel differently up to different work activities. Please state in how far you agree with the following statements concerning teaching”. The first sentence was employed in order to establish a sense of openness and honesty, as the following items concern self-assessments of ability, which require the willingness to scrutinize oneself critically. All items are rated on a five-point Likert-scale of agreement from 0 = “does not apply at all” to 4 = “applies completely”. All items that involve teaching in the presence of a patient were presented at the end of the questionnaire proceeded by the words: “Please only give your rating concerning the following statements if you are involved in teaching with patients (e.g. bedside teaching).”

In order to analyse the response process and to detect problems before data collection, a cognitive debriefing was conducted with three internists working at the Department of Internal Medicine at the University Medical Center Hamburg-Eppendorf. During the first phase, participants filled out the preliminary questionnaire and discussed the comprehensibility of the items afterwards. All items were perceived as comprehensible. In the second phase, the construct of self-efficacy and its underlying theory were explained and the participants were asked about the items’ representativeness with respect to their teaching situations. As no suggestions for changes or additions were made, all items were left in their original wordings. The PTSQ questionnaire can be found in Additional file 1.

### Further instruments and materials

#### Physician teaching motivation questionnaire (PTMQ)

The PTMQ is a validated questionnaire measuring teaching motivation based on SDT and comprising the subscales intrinsic, identified, introjected and external teaching motivation as well as teaching amotivation. It has been successfully validated on the same sample as the PTSQ, confirming its factorial, convergent, criterion and incremental validity as well as good internal consistencies for all subscales scales except for one with an acceptable internal consistency [37].

#### Perceived teaching involvement (PTI)

We defined PTI as the endeavour to use personal behavioural and cognitive resources actively in order to provide high quality teaching. Based on this definition, we created 15 statements about engaging behaviourally and/or cognitively in teaching before, during and after lessons, indicating efforts to provide high quality teaching, e.g.: “I try to prepare each lesson carefully” and “It is very important for me to provide good teaching”. Our indicators of PTI where derived from research on good clinical teachers [36] and complemented by behavior identified within our own comprehensive practical experience in medical teaching. A five-point Likert-scale of agreement was used for the rating of these items. In the sample of this study, the PTI scale showed a good internal consistency (Cronbach’s α = .87).

#### Teaching experience

SCT postulates four sources of self-efficacy: enactive mastery experiences, vicarious experiences, verbal persuasion, and the subjective interpretation of physiological and affective states during an action. Enactive mastery experiences, which can be defined as situations in which an individual feels to have succeeded in completing a task, constitute the most important influence [38]. Therefore, we assumed that teaching self-efficacy is positively correlated with teaching experience and asked teachers to state their teaching experience in years. As for further socio-demographic characteristics, we gathered data on age, gender, occupational position, medical specialty, occupational position and status of postdoctoral lecture qualification. Inclusion criteria were fulfilled if the participants were working as full-time physicians in the setting of a German University Medical Center, if they taught in the subjects of internal medicine or surgery and if they were mostly involved in bedside teaching (BST). BST covers a large part of clinical teaching in the presence of patients in Germany with internal medicine and surgery being the most extensively taught subjects. The final paper-and-pencil questionnaire was distributed to 645 physicians from six German University Medical Centers that were chosen due to their large number of students and as they represent different regions of Germany. At German University Medical Centers, attending physicians, consultants, and all residents are involved in clinical teaching independently of their intended career paths. Data collection in this cross-sectional study took place from March 2014 until July 2014.

### Data analysis

#### Data preparation

All Likert-type scales were treated as interval scales [39]. If at least 90 % of the items in the respective scale per participant were present, missing values in the PTSQ, the PTMQ and for PTI scales were replaced using the EM-algorithm in SPSS for factor analyses. When the proportion of answered items was lower, the questionnaires were excluded from the respective calculations.

#### Item selection and factorial validity

In order to select the items and to explore the factorial structure of the PTSQ, we first performed exploratory structural equation modelling (ESEM) with Mplus version 7.2 [40]. ESEM aims at exploring the multidimensionality of constructs while omitting problems of reliance in the more restrictive conventional confirmatory factor analysis (CFA) [41]. As we assumed that all potential second-order factors would constitute a part of a first-order factor and therefore would be correlated with each other, we chose the oblique geomin rotation method [42]. Models with one, two, three and four correlated factors were obtained. We expected at least one separate factor for all items referring to teaching with patient participation, but made no assumptions about the factorial structure of the remaining items. The chosen model with an additionally assumed higher-order general teaching self-efficacy factor was tested with conventional CFA in SPSS AMOS version 22. Correlations between error variances were restricted to be zero. Descriptive investigation of the responses to each item showed that none of the values for skewness (range from −0.74 till 0.26) or kurtosis (range from −0.06 till 2.26) exceeded those values recommended for the assumption of univariate normal distribution for CFA [43]. To assess global model fit, the Root Mean Square Error of Approximation (RMSEA), the Standardized Root Mean Square Residual (SRMR), the Tucker-Lewis Index (TLI) and the Comparative Fit Index (CFI) were calculated. Recommended expert cut-off-values for the RMSEA range from < 0.05 to < 0.08, for the SRMR from < 0.05 to < 0.08 and for the CFI and the TLI from ≥ 0.95 to ≥ 0.80 (stricter recommendations presented first) [44]. As for local goodness-of-fit, factor loadings and factor-scale congruence, calculated as Pearson correlations between the factor scores and their corresponding scale sums [45] and indicating how precisely the latent factor is captured by the simple sum of the item responses on the corresponding scale, were assessed.

Furthermore, we explored whether a shorter version of the PTSQ measuring only general teaching self-efficacy would display similar goodness-of fit, and if so, whether it also meets the other validity criteria.

#### Scale characteristics

We calculated Cronbach’s alpha’s as indicator of internal consistency as well as means, standard deviations and intercorrelations of all scales. Internal consistencies were evaluated using the recommendations according to Kline (α ≥ 0.9 = excellent; 0.7 ≤ α < 0.9 = good; 0.6 ≤ α < 0.7 = acceptable; 0.5 ≤ α < 0.6 = poor; α < 0.5 = unacceptable) [46].

#### Criterion validity

We calculated bivariate Pearson’s correlations of all PTSQ scales with the PTMQ scales, PTI and the teaching experience in years.

## Results

### Sample

Two-hundred forty seven questionnaires of the 645 that were originally distributed were returned, resulting in a response rate of 38.3 %. Four questionnaires were excluded from analysis because less than 90 % of the PTSQ items were answered. Five other questionnaires were excluded because the items involving teaching with patient participation were not answered. As shown in Table 1, the majority of our participants were male, residents and from departments of internal medicine.

**Table 1:** Characteristics of the study sample (*n* = 238)

Age (Years) M (SD)	Sex % (*n*)		Medical specialty % (*n*)		Teaching experience (Years) M (SD)	Occupational position % (*n*)		Postdoctoral lecture qualification % (*n*)	
37.19 (7.83)	female male	30.4 (72) 69.6 (166)	internal medicine surgery n/a	64.4 (153) 35.2 (84) 0.4 (1)	8.77 (7.46)	resident consultant attending physician other	50.8 (121) 12.3 (29) 33.6 (80) 3.3 (8)	yes no n/a	27.1 (64) 72.5 (173) 0.4 (1)

### Factorial structure

As outlined in Table 2, ESEM indicated the best fit for a four-factor-solution. However, as this factor solution was not sufficiently interpretable, we selected the three-factor-solution, displaying good to acceptable global fit (RMSEA = .058, SRMR = .033, CFI = .958, TLI = .933). The following factors for the PTSQ were identified: 1) self-regulation self-efficacy, 2) dyadic regulation self-efficacy and 3) triadic regulation self-efficacy. Self-regulation self-efficacy encompasses items with challenges that focus on potential affective and cognitive threats to a teacher and thus require self-directed regulative counter-measures. Dyadic regulation self-efficacy encompasses items with challenges coming from the interaction between teacher and students and which mainly require didactic, but also affective and motivational interventions directed at the students. Finally, triadic regulation self-efficacy encompasses those items that present challenges emanating from the inclusion of a patient and require cognitive and behavioural regulations of the teacher-student-patient interaction to ensure effective learning. The factors respectively scales and their corresponding items are provided in Table 3.

**Table 2:** Global goodness-of-fit indicators I. for the ESEM models and II. the final CFA models

	RMSEA	SRMR	CFI	TLI
I. ESEM				
*One factor*	0.093	0.063	0.851	0.828
*Two factors*	0.077	0.048	0.911	0.880
*Three factors*	0.058	0.033	0.958	0.933
*Four factors*	0.044	0.026	0.980	0.962
II. CFA				
*Three factors with general factor*	0.055	0.048	0.948	0.939
*Short version*	0.000	0.014	1.000	1.007

*Abbreviations*: *TLI* Tucker-Lewis-Index, *RMSEA* Root Mean Square Error of Approximation, *SRMR* Standardized Root Mean Square Residual, *CFI* Comparative Fit Index

**Table 3:** PTSQ long version and short version items, means, standard deviations, skewness and kurtosis

Instruction: “One can feel differently up to different work activities. Please state in how far you agree with the following statements concerning teaching”.				
Item		M (SD)	Skewness	Kurtosis
Self-regulation self-efficacy				
1	Even if students ask difficult questions, I am able to answer them correctly.	2.84 (0.66)	−0.23	0.66
2	Even if I am under time strain, I am able to concentrate and provide a well-structured lesson.	2.83 (0.80)	−0.49	0.45
3	Even if I am interrupted during my lesson, I do not grow confused.	2.94 (0.75)	−0.45	0.10
6	*Even if I am in a bad mood or feel stressed, I give a good lesson.*	2.62 (0.77)	−0.71	1.22
7	*Even if I am assigned to a lesson at very short notice, I give a good lesson.*	2.74 (0.79)	−0.79	1.43
11	Even if I get annoyed about the students’ behaviors or appearance, I am able to give a good lesson.	2.60 (0.75)	−0.36	0.49
Dyadic regulation self-efficacy				
4	*Even if students seem tired or demotivated, I manage to make them enthusiastic about the lesson.*	2.34 (0.68)	0.26	0.96
5	I am able to integrate even the weakest students into the lesson.	2.23 (0.76)	0.18	0.64
8	Even if I am faced with big student groups, I reach every student.	2.02 (0.87)	−0.19	0.16
9	If new didactic concepts are introduced by the deanery or other instances it is easy for me to implement them.	2.14 (0.83)	0.04	0.34
10	*I am very good at adapting to different degrees of prior knowledge in a student group.*	2.56 (0.77)	−0.09	−0.06
Triadic regulation self-efficacy				
12	*Even if it is difficult for me to make an unambiguous diagnosis for a certain patient, I can still provide a lesson from which the students benefit.*	3.21 (0.62)	−0.50	1.02
13	*Even if a patient shows a difficult conduct, I provide a good lesson.*	3.03 (0.65)	−0.60	1.90
14	Even if no patient is available who fits to the learning goals I am able to make good use of the lesson.	3.10 (0.65)	−0.74	2.26
15	I am a very good role model for the students in dealing with patients.	3.06 (0.62)	−0.14	0.00
16	Even if a patient hardly speaks German, I can equip the student with important knowledge.	2.91 (0.78)	−0.65	0.98

Items of the PTSQ short version are presented in *italics*

**Fig. 1:**
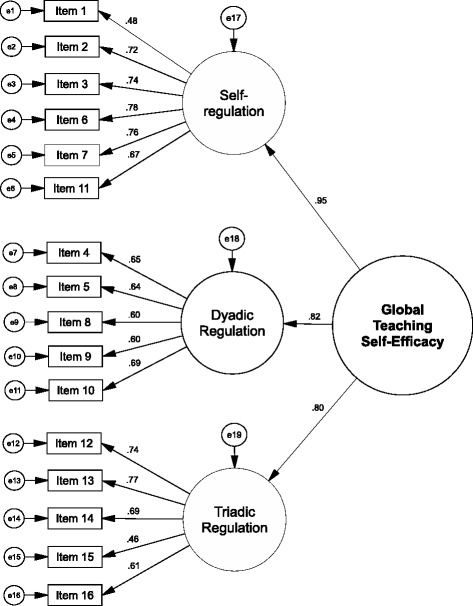
Factorial structure of the PTSQ

**Fig. 2:**
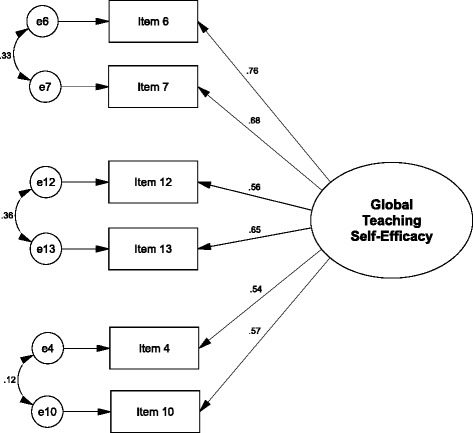
Factorial structure of the PTSQ short version

In a next step, we performed a CFA with these three factors and a superordinate general teaching self-efficacy factor. As can be seen in Table 2, global goodness-of-fit indicators demonstrated a good fit of the model to the data (RMSEA = .055, SRMR = .048, CFI = .948, TLI = .939). Regarding local fit, all factor loadings were higher than 0.4 (Fig. 1), and all factor-scale congruence estimates displayed excellent values nearing a correlation coefficient of one, except for the PTSQ triadic regulation subfactor that displayed a good congruence (Table 4). For a potential short version of the PTSQ, we specified a CFA model with each of the two items with the highest loadings on the three subfactors of the PTSQ long version, allowing for correlated error variances between each item pair belonging to the same factor of the long version. This resulted in a six-item scale. As shown in Table 2, all global indicators demonstrated excellent fit of the model to the data (RMSEA = .000, SRMR = .014, CFI = 1.000, TLI = 1.007), and factor loadings above 0.4 (Fig. 2) as well as a factor-scale congruence close to one indicated excellent local fit (Table 4). Therefore, we decided to validate this version of the PTSQ further, subsequently called “PTSQ short”.

**Table 4:** Means, standard deviations, factor-scale congruence, intercorrelations and internal consistency of the PTSQ, its subscales and the PTSQ short version

Scale	M	SD	Factor-scale congruence	Scale correlations				
				1	2	3	4	5
1. PTSQ global	2.70	0.46	.977**	(.90)	.903**	.838**	.810**	.946**
2. PTSQ self-regulation	2.76	0.57	.980**	.903**	(.85)	.627**	.622**	.872**
3. PTSQ dyadic regulation	2.27	0.57	.965**	.838**	.627**	(.77)	.515**	.763**
4. PTSQ triadic regulation	3.06	0.49	.902**	.810**	.622**	.515**	(.79)	.774**
5. PTSQ short	2.75	0.52	.992**	.946**	.872**	.763**	.774**	(.82)

Cronbach’s alpha’s are presented in parentheses

*Abbreviations*: *PTSQ* Physician Teaching Self-Efficacy Questionnaire, *PTMQ* Physician Teaching Motivation Questionnaire, *PTI* perceived teaching involvement

*p < .05. **p < .01 (two-tailed)

### Scale characteristics

As can be seen in Table 4, the Cronbach’s alpha for the total score of the PTSQ indicates an excellent internal consistency (α = .90), while the self-regulation subscale (α = .85) and the short version (α = .82) indicate a good internal consistency, and the dyadic (α = .77) and triadic regulation (α = .79) subscales indicate an acceptable consistency. The PTSQ and the PTSQ short showed a high intercorrelation (r = .946) and their means were nearly identical. Among the subscales of the PTSQ-long, triadic regulation had the highest mean, dyadic regulation the lowest. The self-regulation subscale showed the highest correlation with the total score and triadic regulation the lowest, but all subscale correlations were high.

### Concurrent criterion validity

Concerning teaching motivation, the correlation patterns are in accordance with SDT for the most part (Table 5). All PTSQ scales as well as the PTSQ short showed their highest significant positive correlations with intrinsic teaching motivation followed by identified teaching motivation, except for the triadic regulation subscale. All PTSQ scales as well as the PTSQ short showed their highest significant negative correlations with teaching amotivation followed by external teaching motivation, except for the triadic regulation subscale, which did not correlate with external teaching motivation significantly. The PTSQ dyadic regulation subscale and the PTSQ short showed no significant correlation with introjected teaching motivation.

**Table 5:** Correlations of the PTSQ scales with teaching motivation, perceived teaching involvement and teaching experience

	PTSQ long total	PTSQ long self-regulation subscale	PTSQ long dyadic subscale	PTSQ long triadic subscale	PTSQ short
Teaching motivation (PTMQ)					
Intrinsic	.424**	.322**	.438**	.336**	.380**
Identified	.364**	.232**	.368**	.359**	.330**
Introjected	−.135*	−.138*	−.074	−.131*	−.127
External	−.202**	−.203**	−.181**	−.122	−.175**
Amotivation	−.307**	−.258**	−.297**	−.232**	−.271**
PTI	.498**	.349**	.546**	.395**	.447**
Teaching experience (years)	.209^**^	.177^**^	.156^*^	.214^**^	.182^**^

*Abbreviations*: *PTSQ* Physician Teaching Self-Efficacy Questionnaire, *PTMQ* Physician Teaching Motivation Questionnaire, *PTI* perceived teaching involvement

*p < .05. **p < .01 (two-tailed)

All PTSQ scales as well as the PTSQ short showed significant positive correlations with the PTI of medium to large effect sizes (Table 5). The mean across all items of the PTSQ shared more variance with PTI than the PTSQ short. Among the PTSQ subscales, the dyadic regulation scale showed the highest and the self-regulation subscale the lowest correlation. All PTSQ scales and the PTSQ short showed significant positive correlations with the physicians’ teaching experience in years with small effect sizes. The total score of the PTSQ shared more variance with age than the PTSQ short version. Among the PTSQ subscales, the triadic regulation scale showed the highest and the self-regulation subscale the lowest correlation.

## Discussion

In this study, we developed and validated an instrument measuring physicians’ teaching self-efficacy, the PTSQ, and a corresponding short version. The factorial structure, reliability and concurrent criterion validity support the suitability of the PTSQ and its short version to assess physicians’ teaching self-efficacy. Global and local fit indicators of both versions suggest a good to excellent factorial validity of the instrument. The factor/scale-congruence of the triadic regulation subscale constitutes the only exception and does not indicate an entirely satisfying local fit. ESEM delivered a well interpretable factorial solution for the PTSQ with additional support by CFA. Concerning scale characteristics, Cronbach’s alpha’s indicated acceptable to excellent internal consistency. With regard to criterion validity, all scales showed associations as hypothesized and in accordance with SCT and SDT, with one slight deviation concerning the correlation pattern of the triadic regulation subscale with the PTMQ scales.

With respect to global teaching self-efficacy, the PTSQ is superior to its short version in terms of reliability and its sensitivity to detect relationships with other constructs derived from theory. It shows a higher internal consistency and consistently higher correlations with all constructs used in this study for assessing construct validity. However, the short version displays an excellent factorial structure as indicated by global and local fit indicators, and its factor-scale congruency showed the highest value among all scales. Furthermore, the means for global teaching self-efficacy as measured by both versions of the PTSQ were almost identical in this sample and the correlation between both scores was high. This indicates that an individual physician will receive very similar scores for global teaching self-efficacy measured by either version of the PTSQ.

In recent years, doubts have been raised about the causality and generality of the relationship between self-efficacy beliefs and performance, leading to a re-assessment of this previously assumed linear effect. One concern regards the potential confounding of actual objective abilities and the subjective belief in one’s abilities [47]. Some authors argue that self-efficacy beliefs are a function of past performance and that self-efficacy does not predict performance incrementally when controlled for past performance [47]. However, a study from medical education research suggests that self-efficacy is a unique predictor of performance, even when controlling for an objective measure of ability [1]. Even if subjective self-efficacy beliefs are partly influenced by objective indicators of past performance, self-efficacy will remain useful in situations where objective indicators of past or present performance are unavailable to the researcher or hard to access. Furthermore, studies which imply that a slight overestimation of one’s capabilities has the most favorable effect on motivation and performance suggest that self-efficacy is more than a function of objective indicators and stress the importance of subjectivity [37].

Another concern originates from studies that found an insignificant or a negative impact of self-efficacy on performance [48]. As an explanation, it is assumed that high self-efficacy for a certain task can result in an individual’s assumption that she or he can allocate less resources to this task [48]. This effect seems to be restricted to situations in which no feedback during a task is available. Both arguments underscore the necessity to scrutinize and re-examine these results more closely and future studies will have to elaborate further on these contradictory findings. However, at this point, they stand against many studies, along with several meta-analyses [10–12], that confirm a positive relationship between self-efficacy and performance.

There are several areas of application for the PTSQ. It could be used in research, e.g. concerning influence factors on and consequences of teaching self-efficacy, before teacher trainings to detect difficulties in teaching as well as for internal quality management.

We recommend to interpret the subscale means in relation to each other. For example, in the sample of this study, self-efficacy for dyadic regulation was less pronounced in comparison to the other subscales, and therefore in an adapted training aspects measured by this subscale might be prioritized. The PTSQ short might find its application as a time-efficient instruments for research with clinicians, since time strain is a constant companion in hospital-based physicians’ daily working routine [49–52], and chronic stress seems to be higher for hospitalists than in the general population [53]. In addition, the PTSQ short might help to increase response rates, which have been shown to be lower for physicians than for non-physicians in mail surveys [54]. Furthermore, a negative effect of the word length in questionnaires for physicians on the response rate has been demonstrated [55]. The PTSQ has been validated within a clinical context involving patients and the triadic regulation subscale cannot be applied to other situations. However, it seems plausible that the other two subscales and a global teaching-self-efficacy score encompassing these subscales could be applied to teaching situations without patients such as lectures and seminars.

### Limitations of this study

The strongest limitation of this study lies in the necessity to validate the PTSQ against other scales that had not been validated at the time of data collection, namely the items measuring perceived teaching involvement and the PTMQ scales, which had been validated in the same sample as the PTSQ. However, there was no other option, as our initial literature research yielded no results for instruments measuring teaching motivation and teaching involvement that are applicable to and specific enough for the working context of teaching hospital-based physicians. Another limitation of this study might arise from the cross-sectional, self-reported data we used, which bears the risk of common method variance (CMV) issues [56]. While some authors argument that the problem of CMV is exaggerated [56], others doubt the accuracy of statistical control techniques [57]. Motivation is a construct that can only be assessed by introspection and self-report. However, teaching involvement could be operationalized more objectively in future studies, as it encompasses a strong behavioural aspect. While teaching motivation and teaching involvement might be susceptible to CMV, the teaching experience in years presents a more objective type of data and is still consistent with our assumptions. As participation in this study was voluntary and we cannot exclude a self-selection bias of more motivated and self-efficacious participants, the representativeness of the means we found cannot be guaranteed. The sensitivity to change of the PTSQ could not be assessed in this study; therefore, its application in pre-post evaluations has to be conducted cautiously. However, as instruments measuring specific self-efficacy in distinct domains and for distinct tasks have consistently been shown to be sensitive to change [58–60], we assume that the PTSQ is also suitable for pre-post testing.

### Future research

As our initial validation of both PTSQ versions showed promising results, our instrument could be translated and tested in different languages. Our results regarding criterion validity could be further supported by other research methods apart from self-administered questionnaires, such as student evaluations or teacher observation. Furthermore, the determination of norm values for different populations might help to interpret the values of the PTSQ in a more absolute way rather than the relative way we suggested in the discussion. Cut-off values might help to determine deficiencies in a reliable way. Additionally, the test-retest-reliability of all scales should be investigated, as well as their sensitivity to change due to teacher trainings, curriculum changes and other measures. In this study, teaching involvement was measured using a self-rating instrument. In future studies, teaching involvement should be measured using data that are more objective. One purpose of this instrument is to detect deficiencies in self-efficacy that might lead to reduced teaching quality. While the relationship between self-efficacy and work performance has been generally established, teacher-centred research tailored to the field of medical education is missing. SCT suggests several sources of self-efficacy that can be used to enhance self-efficacy [38]. The PTSQ could help to evaluate whether and to which degree trainings are suitable to enhance teaching self-efficacy in physicians.

## Conclusions

The factorial validity, reliability and criterion validity indicate that both the PTSQ and its short version are well suited to measure teaching self-efficacy in hospital-based physicians. We recommend the PTSQ for assessing global teaching self-efficacy if the different aspects of teaching self-efficacy are relevant for a particular research question. The PTSQ short can be applied when the highest possible response rates are needed in situations that might be time-critical for the targeted physicians.

## Additional file

Additional file 1: PTSQ Questionnaire. (DOCX 19 kb)

## Abbreviations

CFA: Confirmatory factor analysis
CFI: Comparative Fit Index
CMV: Common method variance
ESEM: Exploratory structural equation modelling
PTI: Perceived teaching involvement
PTMQ: Physician Teaching Motivation Questionnaire
PTSQ: Physician Teaching Self-Efficacy Questionnaire
RMSEA: Root Mean Square Error of Approximation
SCT: Social cognitive theory
SDT: Self-determination theory
SRMR: Standardized Root Mean Square Residual
TLI: Tucker-Lewis Index
