# Supplementary material for: Psychometric properties of the newly developed Physician Teaching Self-Efficacy Questionnaire (PTSQ)
Source: BMC Med Educ. 2016 Sep 22;16:247. doi: 10.1186/s12909-016-0764-4 (PMC5034456; doi:10.1186/s12909-016-0764-4)
Supplement: Additional file 1: — PTSQ Questionnaire. (DOCX 19 kb) [file 12909_2016_764_MOESM1_ESM.docx]

|  | **One can feel differently up to different work activities. Please state in how far you agree with the following statements concerning teaching.** | | | | | |
| --- | --- | --- | --- | --- | --- | --- |
|  |  | does not apply at all | does rather not apply | partially applies | largely applies | fully applies |
| 1 | Even if students ask difficult questions, I am able to answer them correctly. | 🞎_0_ | 🞎_1_ | 🞎_2_ | 🞎_3_ | 🞎_4_ |
| 2 | Even if I am under time strain, I am able to concentrate and provide a well-structured lesson. | 🞎_0_ | 🞎_1_ | 🞎_2_ | 🞎_3_ | 🞎_4_ |
| 3 | Even if I am interrupted during my lesson, I do not grow confused. | 🞎_0_ | 🞎_1_ | 🞎_2_ | 🞎_3_ | 🞎_4_ |
| 4 | *Even when the students seem tired or demotivated, I manage to get them enthusiastic about the lesson.* | 🞎_0_ | 🞎_1_ | 🞎_2_ | 🞎_3_ | 🞎_4_ |
| 5 | I am able to integrate even the weakest students into the lesson. | 🞎_0_ | 🞎_1_ | 🞎_2_ | 🞎_3_ | 🞎_4_ |
| 6 | *Even when I am in a bad mood or feel stressed, I give a good lesson.* | 🞎_0_ | 🞎_1_ | 🞎_2_ | 🞎_3_ | 🞎_4_ |
| 7 | *Even when I am assigned to a lesson at very short notice, I give a good lesson.* | 🞎_0_ | 🞎_1_ | 🞎_2_ | 🞎_3_ | 🞎_4_ |
| 8 | Even when I am faced with big student groups, I reach every student. | 🞎_0_ | 🞎_1_ | 🞎_2_ | 🞎_3_ | 🞎_4_ |
| 9 | When new didactic concepts are prescribed by the deanery or other instances it is easy for me to implement them. | 🞎_0_ | 🞎_1_ | 🞎_2_ | 🞎_3_ | 🞎_4_ |
| 10 | *I am very good at adapting to different degrees of prior knowledge in a student group.* | 🞎_0_ | 🞎_1_ | 🞎_2_ | 🞎_3_ | 🞎_4_ |
| 11 | Even when I get annoyed about the students’ behaviors or appearance, I am able to give a good lesson. | 🞎_0_ | 🞎_1_ | 🞎_2_ | 🞎_3_ | 🞎_4_ |
|  |  |  |  |  |  |  |
| Please also give your rating concerning the following statements if you are involved in **teaching with patients** (e.g. bedside teaching). | |  |  |  |  |  |
| 12 | *Even when it is difficult for me to make an unambiguous diagnosis for a certain patient, I can still provide a lesson from which the students profit.* | 🞎_0_ | 🞎_1_ | 🞎_2_ | 🞎_3_ | 🞎_4_ |
| 13 | *Even when a patient shows a difficult conduct, I provide a good lesson.* | 🞎_0_ | 🞎_1_ | 🞎_2_ | 🞎_3_ | 🞎_4_ |
| 14 | Even when no patient is available fitting to the learning goals I am able to make good use of the lesson. | 🞎_0_ | 🞎_1_ | 🞎_2_ | 🞎_3_ | 🞎_4_ |
| 15 | I am a very good model for the students in dealing with patients. | 🞎_0_ | 🞎_1_ | 🞎_2_ | 🞎_3_ | 🞎_4_ |
| 16 | Even when a patient hardly speaks German, I can equip the students with important knowledge. | 🞎_0_ | 🞎_1_ | 🞎_2_ | 🞎_3_ | 🞎_4_ |

**Physician Teaching Self-Efficacy Questionnaire (PTSQ)**

**Self-regulative self-efficacy = 1,2,3,6,7,11 (items oft the short version: 6,7)**

**Dyadic regulation self-efficacy = 4,5,8,9,10 (items oft the short version: 4,10)**

**Triadic regulation self-efficacy = 12,13,14,15,16 (items oft the short version: 12,13)**
